# Comparison of ultrasonographic features between two types of hepatic echinococcosis and hepatoblastoma in children

**DOI:** 10.3389/fped.2025.1458649

**Published:** 2025-01-28

**Authors:** Yi Tao, Hanqing Kong, Jiawu Li, Zhizhi Tan, Jun Wang, Yan Luo

**Affiliations:** ^1^ Department of Medical Ultrasound, West China Hospital of Sichuan University, Chengdu, Sichuan, China; ^2^ Department of Medical Ultrasound, Shandong Provincial Hospital Heze Hospital, Heze, Shandong, China

**Keywords:** hepatic echinococcosis, hepatoblastoma, ultrasound, contrast-enhanced ultrasound, children, pediatrics, diagnosis

## Abstract

**Introduction:**

The prevalence of hepatic alveolar echinococcosis (AE) and cystic echinococcosis (CE) is notably high among children in western China, with the mortality rate for untreated children potentially reaching up to 90%. Meanwhile, hepatoblastoma is the most common malignant liver tumor in children, yet its treatment differs significantly from that of hepatic echinococcosis. This study aimed to compare and analyze the ultrasonographic and contrast-enhanced ultrasound (CEUS) characteristics of hepatic CE, AE, and hepatoblastoma in children, providing more imaging evidence for accurate clinical diagnosis.

**Methods:**

A retrospective analysis was conducted on preoperative data from children with pathologically confirmed hepatic echinococcosis in our hospital between 2012 and 2024. Furthermore, children consecutively diagnosed with hepatoblastoma between 2020 and 2024, confirmed by histopathological examination, were selected as the control group. Clinical data, conventional ultrasound images, and CEUS characteristics of hepatic echinococcosis and hepatoblastoma were analyzed and compared.

**Results:**

The mean ages of 22 children with hepatic CE and nine children with hepatic AE were 11.6 ± 2.8 years and 11.8 ± 3.6 years, respectively. The mean age of 36 children with hepatoblastoma was 2.9 ± 3.0 years. Significant differences were observed in age and history of residence or travel to endemic areas among children with hepatic echinococcosis and hepatoblastoma (*P* < 0.001). Hepatic CE and hepatoblastoma showed a higher proportion of cystic degeneration [≥50% (54.5%, 12/22), and between 0% and <50% (47.2%, 17/36, respectively)], while hepatic AE predominantly showed no cystic degeneration (88.9%, 8/9). Clear boundaries were most commonly seen in hepatic CE lesions (95.5%, 21/22), while unclear boundaries were more frequent in hepatic AE lesions (88.9%, 8/9) (*P* < 0.05). Calcification was more prevalent in hepatic AE compared to hepatic CE and hepatoblastoma (*P* < 0.05). Hepatoblastoma exhibited richer color Doppler signals (94.4%, 34/36) compared to hepatic CE and AE (*P* < 0.05). CEUS was performed on two hepatic echinococcosis and nine hepatoblastoma lesions. On CEUS, one hepatic AE lesion showed peripheral hyperenhancement in the arterial phase, while one hepatic CE lesion showed no significant enhancement. In hepatoblastoma, nine lesions demonstrated hyperenhancement in the arterial phase and hypoenhancement in the late phase.

**Conclusion:**

This study demonstrates the value of ultrasound in differentiating hepatic echinococcosis from hepatoblastoma in children. Hepatic CE typically manifests as a well-defined cystic or cystic-solid mass, while hepatic AE often presents as an ill-defined cystic-solid or solid mass with diffuse calcifications. Conversely, hepatoblastoma appears as a partially well-defined cystic-solid or solid mass with abundant color Doppler signals within and around the lesion.

## 1 Introduction

Echinococcosis, a zoonotic parasitic disease, primarily manifests as cystic echinococcosis (CE) caused by *Echinococcus granulosus* and alveolar echinococcosis (AE) caused by *Echinococcus multilocularis* (1, 2). Up to 98% of AE lesions and 65%–80% of CE lesions occur in the human liver (1, 3–5). Western China is notably a endemic region for both types of hepatic echinococcosis, accounting for 91% of newly diagnosed cases of hepatic AE globally, particularly among children (1, 2, 6). A survey indicated an incidence rate of 0.8% for CE and 1.3% annually for AE among children in this region (7). If untreated, the mortality rate for hepatic AE can be as high as 90% (3, 5). Treatment approaches include total cystectomy for CE and radical resection or ex vivo resection with auto-transplantation for AE (1, 3, 8).

Early detection and accurate diagnosis of hepatic echinococcosis are crucial for improving the survival rate of affected children. Since most children remain asymptomatic or are unable to describe their symptoms accurately, imaging examinations are pivotal for diagnosis. Furthermore, serology can confirm imaging findings (2, 7). Ultrasound, computed tomography (CT), and magnetic resonance imaging (MRI) are commonly used for the differential diagnosis of abdominal masses in children (3, 5, 8–11). However, due to remote locations and limited medical resources in western China, patients with hepatic echinococcosis face difficulties in obtaining MRI scans, CT scans, and laboratory tests.

Furthermore, these imaging modalities have limitations: CT scanning increases radiation exposure risks for children, while MRI examinations are time-consuming and costly. Conversely, ultrasound is recommended as the preferred method for screening hepatic echinococcosis in endemic regions due to its cost-effectiveness, convenience, and accuracy. It is also essential for intraoperative localization and long-term postoperative follow-up (2, 3, 5, 12).

Hepatoblastoma, the most common malignant liver tumor in children, accounts for 67% of all pediatric liver tumors in children (10, 13, 14). Multidisciplinary standard treatment for hepatoblastoma is mainly surgery combined with chemotherapy. For patients promptly diagnosed, the overall 5-year survival rate can approach 80% (10, 15).

Given the significant differences in treatment approaches, distinguishing between the two types of pediatric hepatic echinococcosis and hepatoblastoma is crucial. The aim of this study was to summarize and compare the clinical characteristics and ultrasonographic manifestations of hepatic echinococcosis and hepatoblastoma, providing additional imaging evidence for differential diagnosis of hepatic echinococcosis in endemic regions.

## 2 Materials and methods

### 2.1 Patient selection

This retrospective analysis was approved by the institutional ethics committee of West China Hospital, Sichuan University. The study population comprised children with hepatic CE and AE who underwent ultrasonography in our department between 2012 and 2024, with diagnoses confirmed by histopathology.

Inclusion criteria: (a) Patients aged ≤17 years. (b) Lesions confirmed as either CE or AE through pathological examination. (c) Availability of complete clinical and ultrasonographic data.
Exclusion criteria: (a) Patients who had undergone prior clinical interventions. (b) Patients who had repeat ultrasonography. (c) Patients with poor-quality ultrasound images. (d) Patients with incomplete or missing ultrasound images.

For the comparative analysis, 36 consecutive patients with histopathologically confirmed hepatoblastoma, who visited our hospital between 2020 and 2024, were selected from the database. The clinical data collected included, but were not limited to the patient's gender, age, and history of residence or travel to endemic regions.

### 2.2 Ultrasound examination

This study utilized three ultrasound systems: the iU22 (Royal Philips, Netherlands), LOGIQ E20 (General Electric Healthcare, USA), and Resona 7 (Mindray Medical Solutions, Shenzhen, China). Each system was equipped with a C5-1 or 5-2 MHz convex array probe for routine ultrasound examinations.

Two children with hepatic echinococcosis underwent contrast-enhanced ultrasound (CEUS) following routine ultrasound. Following the guidelines established by European Federation of Societies for Ultrasound in Medicine and Biology (16), real-time, low mechanical index (0.05–0.08) imaging technology was employed for CEUS. SonoVue was used as the contrast agent, administered intravenously in the elbow at a standard dose of 0.03 ml/kg, followed by a 5 ml saline flush. The examination timing started immediately after SonoVue, injection and the procedure was recorded continuously. The CEUS procedure was divided into three phases: arterial phase (0–30 s post-injection), portal venous phase (30–120 s post-injection), late phase (>120 s post-injection).

All CEUS examinations were performed by radiologists with at least five years of experience in abdominal ultrasound.

### 2.3 Imaging analysis

Ultrasound images were independently reviewed by two radiologists with at least five years of experience in the ultrasound of liver diseases. Disagreements were resolved through consensus. All radiologists involved in the review were blinded to the final pathological results. For patients with multiple liver lesions, the most prominent lesion was analyzed.

The conventional ultrasound features evaluated included lesion location, size, number (solitary or multiple), echogenicity (anechoic, hypoechoic, hyperechoic, or mixed echogenicity), echogenicity uniformity (homogeneous or heterogeneous), morphology (regular or irregular), margins (well-defined or ill-defined), composition (cystic, cystic-solid, or solid), cystic degeneration range (none, 0%< and <50%, ≥50%), calcification (absence or presence) and color Doppler signals (absent, rare, or abundant).

The CEUS features evaluated included the enhancement degree of liver lesions compared to the liver background at three different phases (hypoenhancement, isoenhancement, or hyperenhancement); the enhancement pattern of liver lesions during the arterial phase (peripheral rim-like, homogeneous, or heterogeneous enhancement); and the presence or absence of early washout phenomena (<60 s).

This study adopted the standardized CE classification proposed by the WHO-Informal Working Group on Echinococcosis (3, 12), including CE1, CE2, CE3a, CE3b, CE4, and CE5 types. Hepatic AE was classified into six types according to Kratzer et al. (8), which are hailstorm, pseudocystic, ossification, hemangioma-like, metastasis-like, and unclassifiable types.

### 2.4 Statistical analysis

Statistical analyses were conducted using SPSS version 26.0 software (IBM, NY, USA). A *P*-value <0.05 was considered statistically significant. Quantitative data were expressed as mean ± standard deviation, while categorical data were presented as percentages. For comparisons among multiple groups, analysis of variance was used. The Mann-Whitney *U*-test was employed to analyze differences in quantitative data between two groups, such as age distribution and tumor size. Comparisons of categorical variables between two groups were performed using the Chi-square test or Fisher's exact test, as appropriate.

## 3 Results

### 3.1 Clinical data

**Figure 1:**
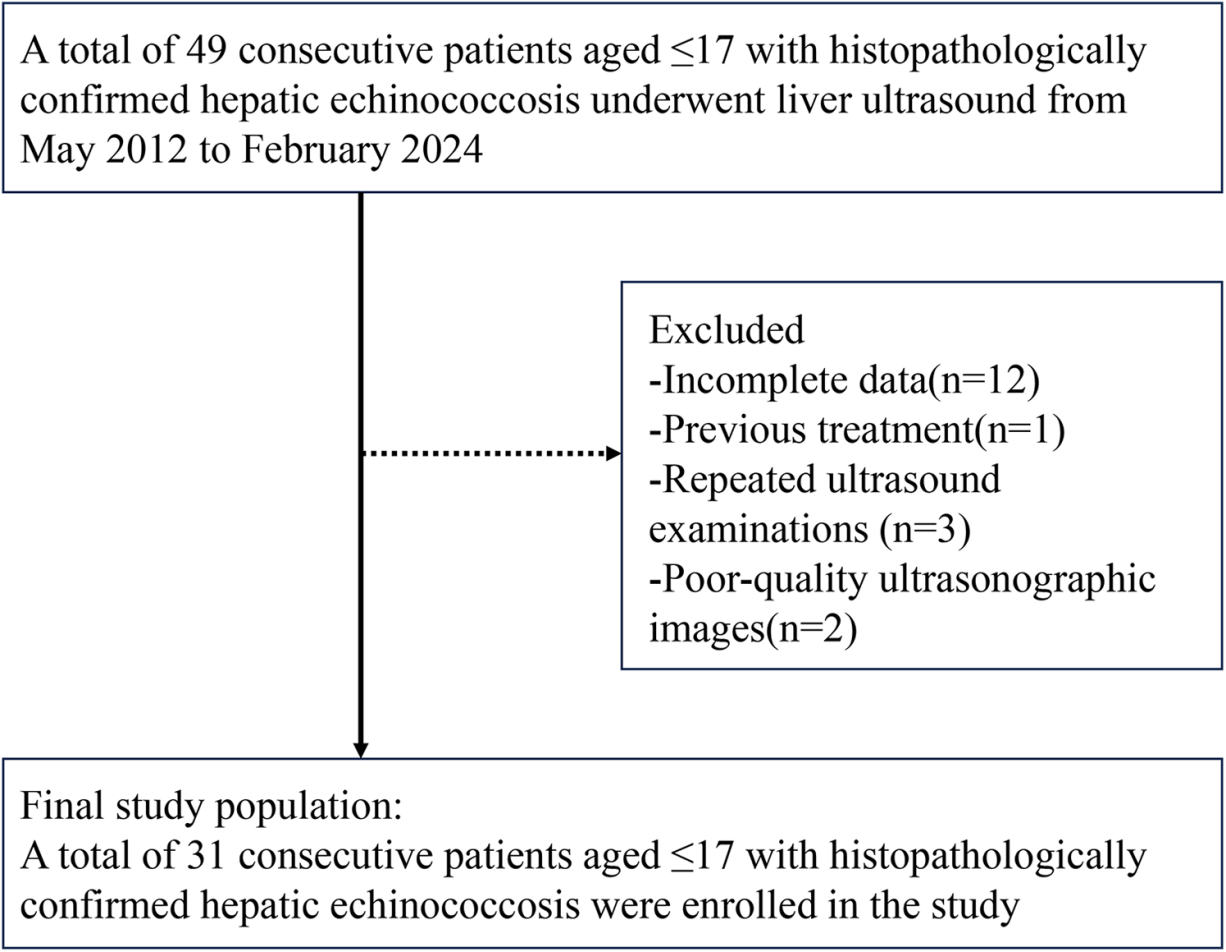
Flowchart showing inclusion of children with hepatic echinococcosis.

A total of 22 patients with pathologically confirmed hepatic CE were included in this study, with a mean age of 11.6 ± 2.8 years. Nine patients with hepatic AE were also included, with a mean age of 11.8 ± 3.6 years. Figure 1 presents the flowchart illustrating the inclusion process for pediatric patients with hepatic echinococcosis. The group of children with hepatoblastoma comprised 19 males and 16 females, with a mean age of 2.9 ± 3.0 years.

The hepatic CE lesions were classified as follows: eight CE1 lesions (36.4%, 8/22), one CE2 lesion (4.5%, 1/22), two CE3a lesions (9.1%, 2/22), five CE3b lesions (22.7%, 5/22), four CE4 lesions (18.2%, 4/22), and two CE5 lesions (9.1%, 2/22). For hepatic AE, the distribution was: two hemangioma-like lesions (22.2%, 2/9), two metastasis-like lesions (22.2%, 2/9), three hailstorm-type lesions (33.3%, 3/9), one ossification-type lesion (11.1%, 1/9), and one pseudocystic-type lesion (11.1%, 1/9).

The mean age of patients with hepatoblastoma (2.9 ± 3.0 years) was significantly younger compared to patients with either type of hepatic echinococcosis (*P* < 0.05). However, no statistically significant difference was observed in gender distribution between patients with the two types of hepatic echinococcosis and those with hepatoblastoma (*P* = 0.313). A comparison of the clinical characteristics between the two types of hepatic echinococcosis and hepatoblastoma in children is presented in Table 1.

**Table 1:** Comparison of clinical characteristics of hepatic CE, AE, and hepatoblastoma in children.

Categories	Hepatic CE (*n* = 22)	Hepatic AE (*n* = 9)	Hepatoblastoma (*n* = 36)	*P*-value
Age	11.6 ± 2.8	11.8 ± 3.6	2.9 ± 3^a^^,^^b^	<0.001
Gender				
Male	14	3	19	0.313
Female	8	6	17	
Residence or travel history to endemic areas				
Yes	19	9	0^a^^,^^b^	<0.001
No	3	0	36	

CE, cystic echinococcosis; AE, alveolar echinococcosis.

^a^ Indicates statistical significance compared to hepatic CE (*P* < 0.05).

^b^ Indicates statistical significance compared to hepatic AE (*P* < 0.05).

### 3.2 Ultrasound findings

In conventional ultrasound, the mean diameter of hepatoblastoma lesions (102.9 ± 25.6 mm, range 41–158 mm) was significantly larger compared to hepatic CE (78.8 ± 26.8 mm, range 39–137 mm) and hepatic AE lesions (67.3 ± 34.7 mm, range 30–126 mm), with statistical significance (*P* < 0.05).

There were significant differences in the composition and cystic degeneration range among hepatic CE, AE, and hepatoblastoma (*P* < 0.001). (a) Hepatic CE lesions had a higher proportion of cystic degeneration range of ≥50% (54.5%, 12/22); (b) Hepatoblastoma had a higher proportion of cystic degeneration range between 0% and <50% (47.2%, 17/36); (c) A higher proportion of hepatic AE lesions (88.9%, 8 out of 9) exhibited no cystic degeneration. (*P* < 0.05).

Margins also differed significantly among hepatic CE, AE, and hepatoblastoma lesions (*P* < 0.05): (a) Well-defined margins were most common in hepatic CE lesions (95.5%, 21/22); (b) Ill-defined margins were most common in hepatic AE lesions (88.9%, 8/9). Calcification was more prevalent in hepatic AE lesions compared to hepatic CE and hepatoblastoma lesions (*P* < 0.05). Color Doppler signals were more abundant in hepatoblastoma lesions (94.4%, 34/36) compared to hepatic CE and AE lesions (*P* < 0.05).

However, there were no significant differences in the number, location, morphology, echogenicity, and echogenicity uniformity among hepatic CE, AE, and hepatoblastoma lesions (*P* > 0.05). A detailed comparison of the conventional ultrasound characteristics of hepatic CE, AE and hepatoblastoma in children is shown in Table 2.

**Table 2:** Comparison of ultrasound characteristics of hepatic CE, AE, and hepatoblastoma in children.

Categories	Hepatic CE (*n* = 22)	Hepatic AE (*n* = 9)	Hepatoblastoma (*n* = 36)	*P*-value
Location				
Right liver	13	3	12	0.218
Left liver	1	2	2	
Caudal lobe/whole liver	8	4	22	
Size (mm)	78.8 ± 26.8	67.3 ± 34.7	102.9 ± 25.6^a^^,^^b^	<0.001
Number				
Solitary	17	7	30	0.836
Multiple	5	2	6	
Morphology				
Regular	11	1	10	0.072
Irregular	11	8	26	
Margins				
Well-defined	21	1^a^	12^a^	<0.001
Ill-defined	1	8	24	
Composition				
Cystic	7			<0.001
Cystic-solid	14	1^a^	17^a^	
Solid	1	8	19	
Cystic degeneration range				
None	2	8^a^	19^a^	<0.001
0%< and <50%	8	1	17	
≥50%	12			
Echogenicity				
Anechoic	6			0.250
Hypoechoic	2	5^a^	9^a^	
Hyperechoic		3	11	
Mixed echoic	14	1	16	
Echogenicity uniformity				
Homogeneous	5		6	0.310
Heterogeneous	17	9	30	
Calcification				
Absence	15	1^a^	26^b^	0.002
Presence	7	8	10	
Color Doppler signals				
Absent	19	7		<0.001
Rare	3	2^a^	2^a^^,^^b^	
Abundant			34	

CE, cystic echinococcosis; AE, alveolar echinococcosis.

^a^ Indicates statistical significance compared to hepatic CE (*P* < 0.05).

^b^ Indicates statistical significance compared to hepatic AE (*P* < 0.05).

**Figure 2:**
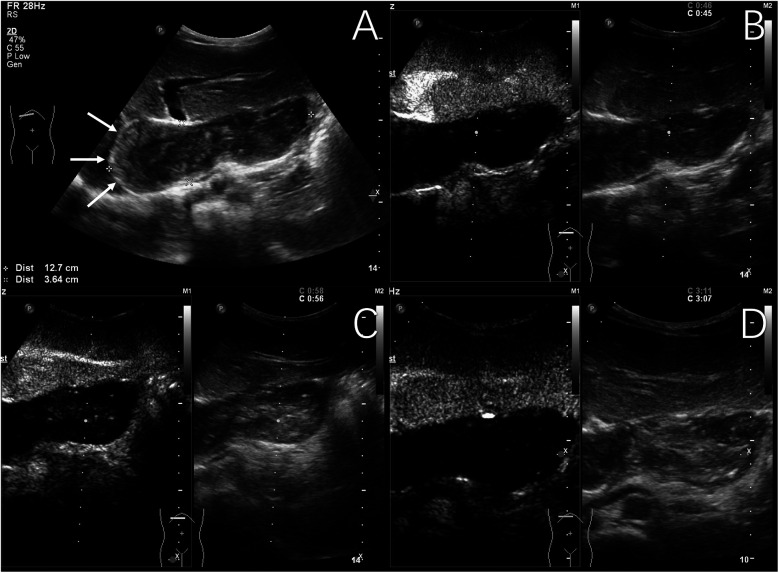
Typical ultrasound findings of hepatic cystic echinococcosis (case 1). Ultrasound of the liver revealed several mixed echogenic masses, with the larger one located in the caudate lobe of the liver, protruding between the left liver and stomach, with a maximum diameter of 127 mm, clear margin, irregular morphology, and patchy strong echoes detected around it (The white arrows show eggshell-like calcification around the lesion) **(A)** contrast-enhanced ultrasound examination showed no significant enhancement during the arterial phase **(B)**, portal venous phase **(C)**, and late phase **(D)**.

**Figure 3:**
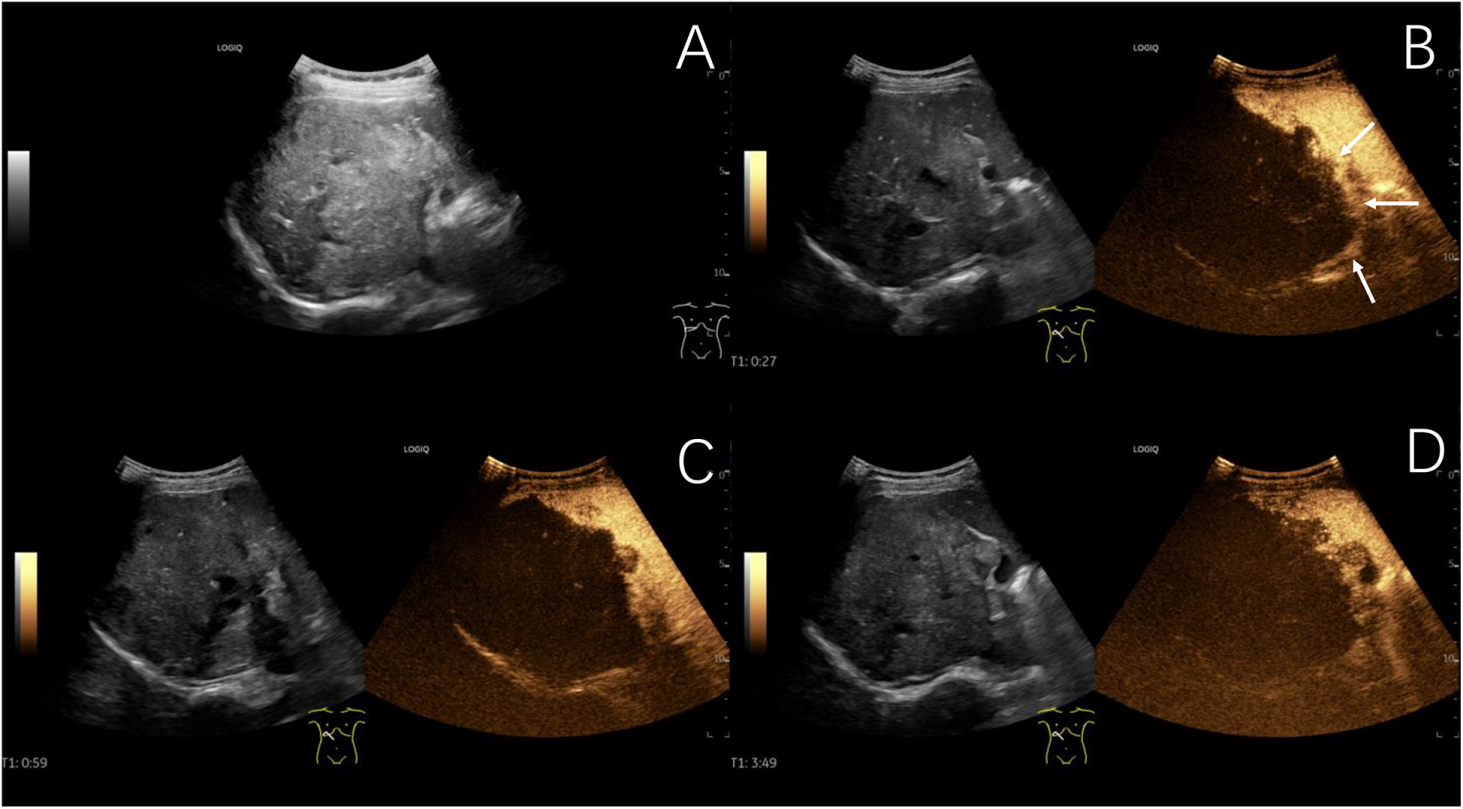
Typical ultrasound findings of hepatic alveolar echinococcosis (case 2). Ultrasound showed a 101 mm hyperechoic mass in the right liver, with unclear boundaries, irregular shape, and patchy strong echoes inside **(A)** contrast-enhanced ultrasound examination showed slight peripheral enhancement during the arterial phase **(B)**, with no enhancement observed internally in the arterial phase, portal venous phase **(C)**, or late phase **(D)** (The white arrows indicate the peripheral slightly hyperenhanced sign of the lesion in the arterial phase).

**Figure 4:**
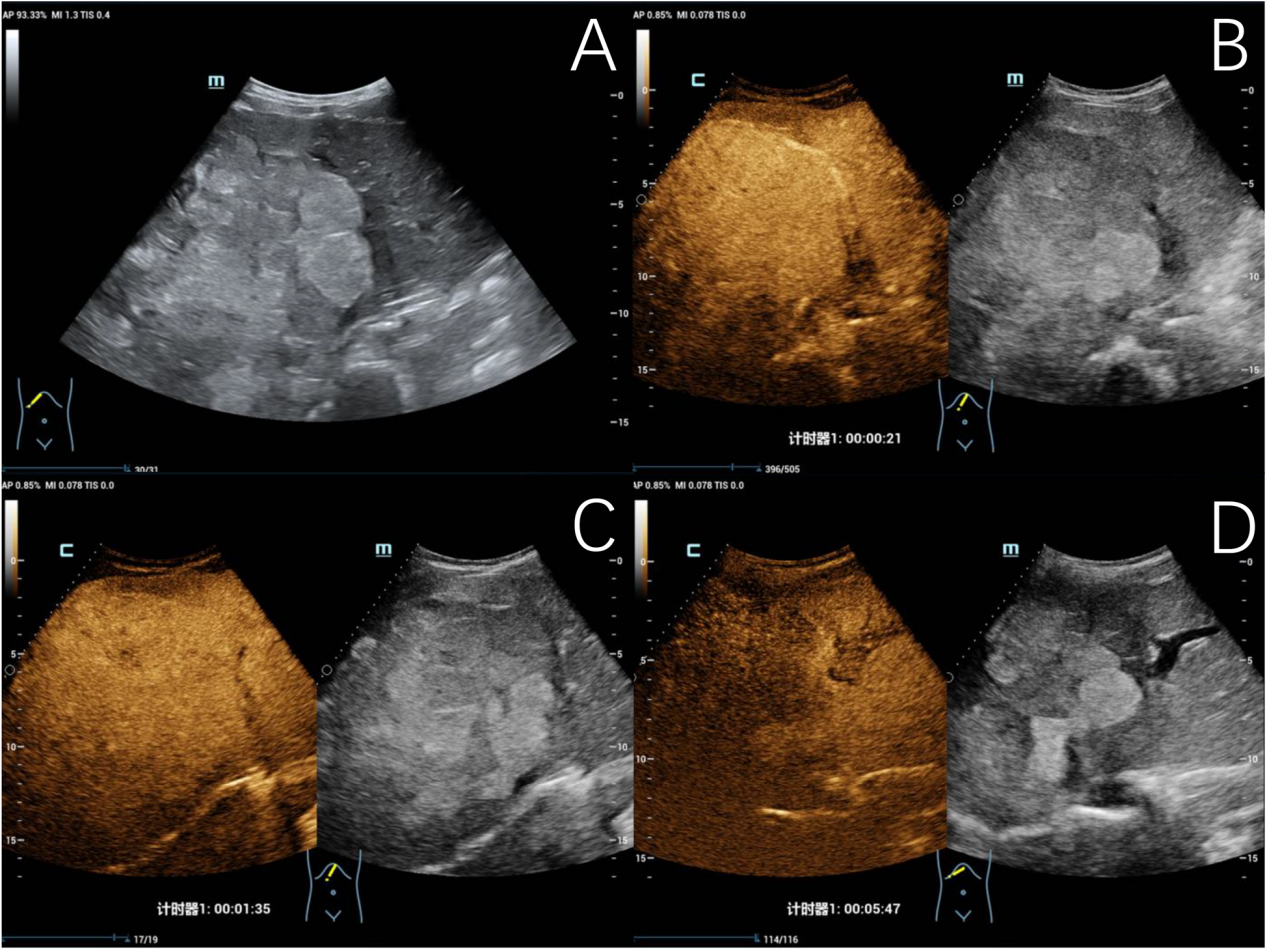
Typical ultrasound findings of hepatoblastoma (case 3). Ultrasound revealed a mixed echogenic mass in the right liver and left inner lobe, with clear boundaries, irregular morphology, and a maximum diameter of 140 mm **(A)** contrast-enhanced ultrasound examination showed heterogeneous hyperenhancement in the arterial phase **(B)**, isoenhancement in the portal venous phase **(C)**, and hypoenhancement in the late phase **(D)**, with patchy non-enhancing areas observed within.

Two out of 31 children of hepatic echinococcosis underwent CEUS. Nine out of 36 children with hepatoblastoma underwent CEUS. One hepatic CE lesion showed no significant enhancement during the arterial, portal venous, and late phases (Figure 2). One hepatic AE lesion showed slight peripheral enhancement during the arterial phase (Figure 3). Nine hepatoblastoma lesions showed hyperenhancement during the arterial phase, six lesions began to washout in the portal venous phase, and nine lesions showed hypoenhancement in the late phase (Figure 4).

## 4 Discussion

Echinococcosis, a zoonotic disease prevalent in the northwestern plateau regions of China, primarily targets the liver (1, 2). Ultrasonography has been extensively used to differentiate adult hepatic echinococcosis from other hepatic tumors (17–19); however, studies in the pediatric population are limited. Notably, hepatoblastoma, a malignancy specific to children, exhibits partial overlapping imaging characteristics with hepatic echinococcosis, posing challenges in differentiation (5, 8). This study compares the clinical and ultrasonographic manifestations of two types of hepatic echinococcosis and hepatoblastoma in children.

In this study, 28 children (90.3%, 28/31) with hepatic echinococcosis had residence or travel history to endemic areas. It has been reported that patients with hepatic echinococcosis typically have history of residence or travel to endemic areas and may become infected through accidental ingestion of parasite eggs by definitive hosts (such as dogs) or intermediate hosts (such as rodents, cattle, and sheep) (2). These eggs hatch into oncospheres upon stimulation by digestive fluids in the gastrointestinal tract, which are then absorbed into the bloodstream and transmitted to the liver via the portal venous system. The average age of children with hepatoblastoma in this study was 2.9 ± 3 years, consistent with another study where 90% of hepatoblastoma lesions occur in children less than five years (14).

The mean sizes of the hepatic CE, AE, and hepatoblastoma lesions included in this study were 78.8 ± 26.8 mm, 67.3 ± 34.7 mm, and 102.9 ± 25.6 mm, respectively. This is likely due to the non-specific early symptoms of these diseases, particularly in children who may have difficulty describing their symptoms, resulting in lesions often being identified at a significant size (7, 13). Studies have shown that patients with hepatic echinococcosis may remain asymptomatic for five to 15 years (3, 4). Upon detection, 70% of hepatic CE lesions have a diameter >100 mm or occupy >70% of the liver volume. When the diameter exceeds 75 mm, the risk of developing biliary fistula increases to 79% (2, 9). Furthermore, rupture and infection of larger CE lesions can lead to severe complications such as anaphylaxis, disseminated implantation, and septic shock (1, 5). Therefore, early screening and accurate diagnosis are crucial.

In this study, 12 lesions of hepatic CE (54.5%, 12/22) exhibited a cystic degeneration range of ≥50%. Among them, there were seven CE1 lesions, one CE2 lesion, one CE3a lesion, and two CE3b lesions. These lesion types can be identified by specific ultrasonographic signs. The CE1 lesions were characterized by the “double-wall sign”, which manifested as a potential space between the inner and outer cyst walls. The CE2 lesions were identified by the “nested cysts/daughter cysts sign”, where multiple daughter cysts were visible within the mother cyst. The CE3a lesions exhibited the “water lily sign”, characterized by the detachment and floating of the inner cyst within the cyst fluid (3, 12).

The diagnostic challenge lies in distinguishing between pediatric hepatic cystic-solid lesions (predominantly solid components) and solid lesions. Aside from specific signs, assessment must also incorporate lesion margins, calcification, and color Doppler signals to aid in diagnosis. Eight hepatic CE lesions (36.4%, 8/22) had a cystic degeneration range between 0% and <50%, including four CE3b and four CE4 lesions. In comparison, one lesion of hepatic AE (11.1%, 1/9) and 17 lesions of hepatoblastoma (47.2%, 17/36) were within this range. The CE3b lesions form due to the collapse of the inner cyst after rupture, allowing cyst fluid to enter between the inner and outer cyst walls, resulting in a cystic-solid mixed echogenicity mass with a “honeycomb-like” structure. CE4 solid lesions exhibit characteristic “cerebral gyrus sign” and “ball of wool sign”, which arise from the solidification of degenerating cysts, absorption of cyst fluid, and folding and contraction of the cyst wall. One case of pseudocystic AE arose from a large lesion with central ischemic necrosis, characterized by an irregular low-echoic liquefactive necrotic area within a heterogeneous mass, resembling a “worm-eaten” or “lava-like” appearance. Hepatoblastoma demonstrates multiple irregular small liquefactive necrotic areas within the mass.

Two lesions (9.1%, 2/22) of hepatic CE had no cystic degeneration, while eight lesions (88.9%, 8/9) of hepatic AE and 19 lesions (52.8%, 19/36) of hepatoblastoma did not exhibit cystic degeneration, further complicating clinical differentiation. This study found statistically significant differences in margins and calcification among hepatic CE, AE, and hepatoblastoma, indicating that these features can aid in differentiation. Notably, 21 hepatic CE lesions (95.5%, 21/22) had clear margins, while eight hepatic AE lesions (88.9%, 8/9) had unclear margins. This phenomenon may be related to the biological behavior of different types of echinococcosis. Hepatic AE grows infiltratively in a budding manner, eroding surrounding tissue structures, resulting in blurred margins with normal liver parenchyma. Hepatic AE may infiltrate the hepatic hilum, bile ducts, and blood vessels (5, 20, 21). Furthermore, hepatic AE can metastasize to other organs like the brain and lungs via lymphatic and hematogenous routes (2, 17). Due to its behavior of local infiltration and distant metastasis, similar to malignant tumors, AE is also known as “parasitic cancer” (17). All eight lesions of hepatic AE presented as heterogeneous echogenic masses with ill-defined margins. In contrast, hepatic CE grows expansively, with a biological behavior similar to benign tumors, thus often exhibiting clear margins (1).

As the lesions grow and inflammatory reactions occur in the body, coupled with inadequate blood supply to the parasitic tissues, calcium salt deposition may occur. These diseases can be further distinguished based on their calcification characteristics. Type CE5 lesions exhibited a special sign known as the “eggshell calcification wall sign”, characterized by flocculent thickened calcification of the cyst wall. Among the eight hepatic AE lesions, the hailstorm type accounted for the largest proportion (37.5%, 3/8), manifested as diffuse hyperechoic regions within heterogeneous masses. One ossification type lesion presented with clustered calcifications within the lesion. Of the ten calcified hepatoblastoma lesions (27.8%, 10/36), only patchy calcifications were observed.

The statistical differences in color Doppler signals also aid in differentiating hepatoblastoma from the two types of echinococcosis (*P* < 0.05). There were three (13.6%, 3/22) hepatic CE lesions and two (22.2%, 2/9) hepatic AE lesions with rare color Doppler signals, both of which were primarily peripheral punctate color Doppler signals. In contrast, 34 lesions (94.4%, 34/36) of hepatoblastoma exhibited rich color Doppler signals, both internally and peripherally.

CEUS may aid in differentiating between the two types of hepatic echinococcosis and hepatoblastoma. For hepatoblastoma, nine cases showed hyperenhancement in the arterial phase and hypoenhancement in the late phase, consistent with Jiang et al. (22). In contrast, one lesion of hepatic CE showed no enhancement in the arterial, portal venous, or late phases, while one lesion of hepatic AE demonstrated only peripheral rim-like hyperenhancement in the arterial phase. Studies have shown that the most common enhancement patterns of hepatic AE lesions were rim-like enhancement zone visible peripherally from the early arterial to late portal venous phases, with no significant enhancement internally, presenting as a “black hole sign” (17, 19). The lack of internal enhancement may be due to its composition of aggregated vesicles, calcification, and necrotic areas. The peripheral rim enhancement is primarily attributed to the presence of a “rim zone” with abundant microvascular supply between the hepatic AE and surrounding normal liver parenchyma (17, 18, 23). The “rim zone” is closely related to lesion activity, and radical resection is crucial for preventing recurrence. Studies have indicated that CEUS assessment of blood supply in hepatic AE lesions correlates with metabolic activity (24). Further development of ultrasound classification focusing on lesion activity is required in future studies.

This study has several limitations. First, it is a single-center retrospective study. Second, selection bias is inevitable, and the high mortality rate among children with hepatic AE may account for the relatively small number of cases. Third, due to the limited number of cases, further comparison of ultrasound and CEUS features of different subtypes of hepatic AE and hepatoblastoma in children was not conducted. Future multi-center studies with larger samples are required.

This study underscores the clinical value of ultrasound features in differentiating between the two types of hepatic echinococcosis and hepatoblastoma, particularly in resource-constrained endemic regions. Hepatic CE lesions typically present as large, well-defined cystic or cystic-solid masses, while hepatic AE lesions commonly present as ill-defined cystic-solid or solid masses with prevalent diffuse calcification. Sparse punctate color Doppler signals may be observed around some hepatic echinococcosis lesions. Comparatively, hepatoblastoma lesions most frequently appear as partially well-defined cystic-solid or solid mass, characterized by patchy calcifications, multiple irregular liquefactive necrotic areas, and relatively abundant color Doppler signals.

## Data Availability

The data analyzed in this study is subject to the following licenses/restrictions: Data set from West China Hospital of Sichuan University. Requests to access these datasets should be directed to taoyi@wchscu.edu.cn.
